# Probiotic supplementation for optimizing athletic performance: current evidence and future perspectives for microbiome-based strategies

**DOI:** 10.3389/fnut.2025.1572687

**Published:** 2025-07-15

**Authors:** Timea Teglas, Zsolt Radak

**Affiliations:** ^1^Research Institute of Sport Science, Hungarian University of Sports Science, Budapest, Hungary; ^2^Faculty of Sport Sciences, Waseda University, Tokorozawa, Japan

**Keywords:** microbiome, sport, performance, probiotics, exercise

## Abstract

The association between microbiota and physical activity is currently a key focus in sports performance research, and the effects of probiotics administration on athletes represent a relatively new area of research. While existing research highlights the promising potential of probiotics, our understanding of how they benefit highly active individuals remains incomplete. Nonetheless, it appears that probiotics have a beneficial effect on mental health, cognitive functions, sleep, gastrointestinal, and upper respiratory symptoms in adult humans. Additionally, the probiotic supplementation and their performance effects of different types of exercise are crucial when building a training program. In most cases, probiotic supplementation is effective in two major types of exercise: probiotics show strain and duration-specific effects both on endurance-based and intermittent-exercise associated sport. The supplementation can reduce inflammatory process activity and stress-related factors, e.g., anxiety, depression, in intermittent exercise-associated sports. In endurance-based sports, probiotics enhanced lipid metabolites, including short-chain and polyunsaturated fatty acids, modulated the maximal oxygen capacity, and reduced gastrointestinal symptoms. Exploring the relationship between probiotics, microbiome, and exercise performance could offer valuable insights for optimizing training techniques and strategies for professional athletes.

## Graphical abstract

Created via Canva.com.

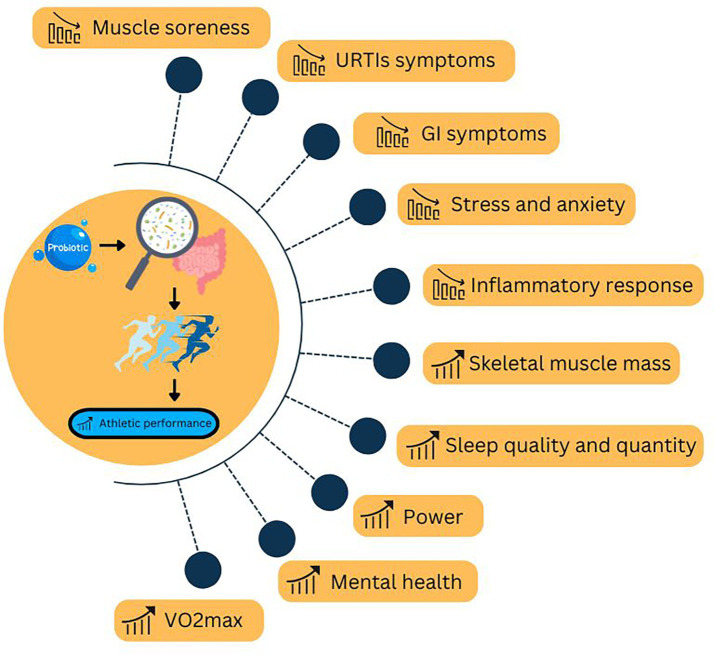

## 1 Introduction

Interest in and knowledge of the gut microbiome have increased drastically in the past 10 years (1, 2). The human gut microbiome represents a complex ecosystem that contributes essential functions to its host (3, 4). Recent large-scale experimental studies have provided evidence of its functional potential. The intricate communities of microorganisms in the human gastrointestinal tract are increasingly recognized as crucial contributors to human health and disease. Some human and *in vitro* studies have demonstrated that the gut microbiome can rapidly respond and change to the diet (5–8). The significance of the gut microbiome is underscored by several vital functions it performs for its human host. These functions include fermenting indigestible food components into absorbable metabolites, synthesizing essential vitamins, detoxifying harmful compounds, outcompeting pathogenic microbes, enhancing the intestinal barrier, and stimulating and regulating the immune system (9–14). In addition, the human gut microbiome plays an important role in biological processes associated with aging (15). Previously described that the microbiome has a crucial role in mental health, and there are correlations between the microbiome and anxiety and trauma-related disorders, and neurodegenerative disorders such as Alzheimer’s (AD) and Parkinson’s diseases (PD) (16–19). Our laboratory previously demonstrated that the microbiome has a critical role in AD, especially in the development of the disease, the mitochondrial function the metabolism-related molecular pathways in the liver (20–22). There is evidence that microbiomes might be a potential therapeutic target for liver diseases (23) and neurodevelopmental disorders, i.e., autism spectrum disorders (23–25).

Probiotics are live microorganisms that provide health benefits to the host when taken in sufficient quantities. Probiotic supplementation has been the research focus for years because it can modify the gut microbiota composition, increasing microbial diversity and supporting the growth and reproduction of health-promoting species (26, 27). While many probiotics can promote overall gastrointestinal, immune system, and brain health, the specific mechanisms through which they act—such as producing bioactive compounds, preventing pathogen adhesion, enhancing gut barrier function, modulating the immune response, and increasing brainwave components—can vary significantly between strains, even within the same bacterial species (28–32). The impact of probiotics on the gut microbiota composition is well-published (33–35). Despite growing evidence, the strain-specific effects of probiotics on athletic performance remain underexplored, necessitating a synthesis to guide personalized interventions.

This review (1) summarizes recent evidence on exercise-induced microbiome changes and (2) evaluates probiotic supplementation’s effects on athletic performance across exercise modalities. To identify relevant literature, studies were sourced from PubMed and Scopus (2015–2024). The search included various keywords and their combinations, such as “probiotics,” “microbiome,” “exercise performance,” and “wheelchair athletes,” focusing on human and animal trials. Articles were selected based on the relevance of their titles, abstracts, and full texts.

## 2 Associations between exercise and microbiome

The microbiota-physical activity association is currently the focus of converging interest in sports performance (36–40). Regular physical exercise provides many health benefits including cardiorespiratory fitness, and it can modify gut microbiota diversity (41, 42). O’Donovan et al. (42) studied 37 athletes from 16 different sports and categorized them based on peak static and dynamic components: low, moderate, and high static components; low, moderate, and high dynamic components. The authors found differences in microbiome and metabolome using shotgun sequencing. In the other study, the 12-week moderate-intensity aerobic exercise (running) showed no significant beneficial effect on the gut microbiota in 224 clinically well adolescents (43). In obese female humans, 6 weeks of cycle ergometer or treadmill training altered gut microbiota composition (44). In another clinical trial, exercise training improves gut microbiota profiles in sedentary people with prediabetes (45). In this study, subjects were randomized to two training modules: sprint and moderate-intensity continuous training for only 2 weeks, and they found training-specific gut microbiota changes. In contrast, short-term high-intensity interval training (HIIT) on a cycle ergometer did not impact the gut bacterial community in overweight men (46). Long-term, chronic exercise improved gut microflora in humans with type 2 diabetes (47). Similar results were observed in the other randomized clinical trial, where the 8-week high-intensity training (HIIT). The relative abundance of *Bifidobacterium, A. municiphila*, and butyrate-producing bacteria such *as Lachnospira eligens, Enterococcus* spp., and *Clostridium Cluster IV* was higher following lower-intensity exercise. In contrast, other butyrate producers (belonging to *Eryspelothrichales and Oscillospirales*) and the methane producer *Methanobrevibacter smithii* were more abundant after higher-intensity exercise (48). In young adolescents, 3 months of moderate-intensity exercise improved depressive symptoms. They increased the relative abundance of *Coprococcus, Blautia, Dorea, Tyzzerella* at the genus level, as well as *Tyzzerella nexilis, Ruminococcus obeum* at the species level (49). In contrast, 6 months of exercise induced subtle changes to the gut microbiota in humans with overweight and obesity (50). In elderly men, a 5-week endurance exercise program also affected the microbiome including a decreased relative abundance of *Clostridium difficile*, and increased *Oscillospira*. These results were correlated with the changes in several cardiometabolic risk factors, such as systolic and diastolic blood pressure (51). Changes in gut microbiome metabolites and redox homeostasis with exercise have been demonstrated in late middle-aged adults with familial and genetic risk for AD (52). In women, Bressa et al. (53) found differences in microbiota profiles between active and passive women. In active women, an increased abundance of health-promoting bacteria (*Bifidobacterium* spp., *R. hominis*, *A. muciniphila*, and *F. prausnitzii*) was present in the microbiota. In older women, 8 weeks of exercise increased the abundance of bacteria associated with an anti-inflammation pathway (54). Our laboratory previously demonstrated that gut microbiome alterations are associated with epigenetic age acceleration and physical fitness (55). We propose an overview of the exercise method or model and the physiological effects with the current evidence (Table 1).

**Table 1:** Summary of associations between physical exercise and microbiome.

Authors	Physical exercise	Physiological effect
O’Donovan et al. (42)	Classification of sports based on peak static and dynamic components	Different microbial diversity in different sports, increased estimated percent of maximal oxygen uptake, increased cardiac output, increased blood pressure load
Wang et al. (43)	12-week running exercise at a moderate intensity of 50–70% HR_max_ for 30 min, 4 days/week	No significant effects
Allen et al. (44)	6-week cycle ergometer or treadmill exercise at a moderate-to-vigorous intensity at 60–75% HR_max_ for 30 to 60 min, 3 days/week	Modulated composition and metabolic capacity of the gut microbiota
Motiani et al. (45)	Sprint and moderate-intensity continuous training for 2 weeks, 3 days/week	Improved VO_2_ peak after sprint, Decreased fatty acid uptake after moderate-intensity continuous training, Reduced systemic and intestinal inflammatory markers; modified microbiota profile in both training modules
Rettedal et al. (46)	Nine sessions of cycle ergometer HIIT on nonconsecutive days over 3 weeks	No significant effects
Pasini et al. (47)	6-months of endurance, resistance, and flexibility training	Modified intestinal microbiota composition and gut barrier function
Wang et al. (49)	3-months moderate intensity for 30 min, 4 days/week	Increased the relative abundance of microbiota genus and species levels, improved depressive symptoms
Torquati et al. (48)	8 weeks of combined aerobic and resistance moderate-intensity continuous training or combined aerobic and resistance high-intensity interval training	Higher relative abundance of *Bifidobacterium*, and few butyrate producers at lower exercise intensity; Higher relative abundance of other butyrate producers and methane producers at higher exercise intensity
Kern et al. (50)	6 months of bike or leisure-time exercise of either moderate (50% of VO_2_peak-reserve) or vigorous intensity (VIG, 70% of VO_2_peak-reserve), 5 days/week	No significant differences between alpha diversity and phenotypical outcomes, Beta diversity changed in all exercise groups
Taniguchi et al. (51)	5-week aerobic exercise on an ergometer, 3 days/week	Changes in the relative abundance of microbiota and correlated with the changes in several cardiometabolic risk factors
Gaitán et al. (52)	26-week treadmill exercise at moderate-to-vigorous intensity, less than 150 min per week	Increased levels of polyunsaturated free fatty acids (PUFAs) and changes in gut microbiome metabolites and redox homeostasis
Bressa et al. (53)	7 days of accelerometer monitoring	Modulated microbiota profile in the active participants: increased abundance of health-promoting bacteria
Zhong et al. (54)	8-week aerobic and resistance exercise for 60 min	Reduced abundance of bacteria associated with pro-inflammation

In animal studies, 12 weeks of resistance training enhanced the diversity of the gut microbiota in rats (56). In the same species, the wheel running exercise for 6 weeks was more effective in early life compared to adult animals (57). In mice, the moderate intensity of physical exercise affected the microbiome profile after 2, 6, 10, and 14 weeks of exercise (58). A similar duration of training (6 weeks) increased *Bifidobacterium* spp. level in exercised mice (59). Another type of exercise, namely swimming exercise, also modulated the relative abundance of the genus *Desulfovibrio*, genus *Streptococcus*, and genus *p-75-a5* in depressed mice after the 5-week training program (60). The impact of physical activity has been demonstrated in neurodegenerative diseases, such as Parkinson’s and Alzheimer’s diseases; the relative abundance of the *Bacteroidetes* was decreased, while *Firmicutes, Actinobacteria, Lactobacillaceae, Streptococcaceae, Lactobacillus, Streptococcus, Lactococcus, Lysinibacillus, Pelomonas,* and *Prevotellaceae_UCG-001* was increased in PD mice (61). In the APP/PS1 transgenic AD mice, 12 weeks of treadmill exercise effectively modulated the gut microbiome profile (62). Yu et al. demonstrated that exercise promoted the growth of butyrate-producing bacteria in the gut and enhanced butyrate production, which in turn enhanced lipid metabolism via the butyrate-SESN2/CRTC2 pathway (63).

## 3 Impact of probiotic supplementation on performance in healthy humans

A few important factors significantly determine sports performance, next to the training methods and strategies, such as sleep quality and quantity, mental health, stress, body composition, gastrointestinal symptoms, URTIs (upper respiratory tract infections) symptoms, and inflammatory responses. As we know, there is a relationship between cognitive function, sport-specific motor skills, and performance (64). Higher cognitive ability is associated with lower psychological distress (65). There is increasing evidence that the gut microbiome correlates with cognitive performance, and probiotic supplementation can improve cognitive functions and performance in healthy adults (33, 66). Probiotic administration mediates neuroprotective effects in healthy elderly (67). In addition, supplementation of *Bifidobacterium longum* BB68S improved cognitive functions such as immediate memory, visual–spatial/constructional memory, language, attention, and delayed memory in healthy older adults (68).

Stress, including anxiety, has been identified as a crucial factor in sport (65, 69). Previously described that sleep affects physical and mental performance, injury risk, recovery, and mental health (70, 71). 4-week probiotic supplementation reduced stress in healthy adults (72). In contrast, Morales-Torres et al. (73) did not find a significant effect after 4 weeks of *Lactobacillus helveticus* R0052 and *Bifidobacterium longum* R0175. The balance of circadian rhythm, i.e., sleep quality and quantity, is important in sports performance (74, 75). In healthy adults, probiotic supplementation is beneficial for cognitive function, mental health, and sleep (33, 66, 76).

*Lactobacillus plantarum* TWK10 significantly elevated exercise performance in a dose-dependent manner and improved the fatigue-associated features correlated with better physiological adaptation in healthy humans (77). Previously described probiotic supplementation showed both a systemic and local reduction of the inflammatory response (78, 79). These results might be important in the reduction of inflammatory responses expected after exercise or exercise-based injury.

URTIs are the most prevalent illnesses among athletes, leading to missed training sessions and competitions. The URTI symptoms, such as runny nose, nasal congestion, sneezing, and sore throat scores can decrease physiological performance (80, 81). Probiotic supplementation can suppress the symptoms of URTIs in adults (82, 83). Relatively short-term *Streptococcus salivarius* K12 supplementation can support the mucosal immune function of active young subjects (84). In contrast, there were no significant changes after probiotic administration in a meta-analysis that included more than 1,500 participants (85).

Endurance sports frequently cause exercise-induced gastrointestinal symptoms, i.e., abdominal bloating, heartburn, and diarrhea in endurance athletes, which may impact the physical and psychological performance (86). In healthy adults, daily supplementation of *Bacillus subtilis* BS50 alleviated gas-related gastrointestinal symptoms after 6 weeks (87). We propose an overview of probiotics and their physiological effects with the current evidence (Table 2).

**Table 2:** Summary of the physiological and performance enhancement effects of probiotic supplementation.

Authors	Probiotics	Performance enhancement effect	Physiological effect	Population
Aljumaah et al. (66)	*Lactobacillus rhamnosus GG*	Cognitive funcions	*Prevotella ruminicola, Bacteroides thetaiotaomicron*, and *Bacteroides xylanisolvens* as taxa correlated with MCI (mild cognitive impairment)	healthy elderly
Kim et al. (33)	*Bifidobacterium bifidum* BGN4 and *Bifidobacterium longum* BORI	Mental flexibility and alleviating stress	Inflammation-causing gut bacteria were significantly reduced, and increased serum BDNF level	healthy elderly
Kim et al. (67)	*Bifidobacterium bifidum* BGN4 and *Bifidobacterium longum* BORI	Neuroprotective effects	Changes in the microbiota-related bile acid metabolism, which can reduce neuroinflammation in microglial cells	healthy elderly
Shi et al. (68)	*Bifidobacterium longum* BB68S	Cognitive functions	Significantly decreased the relative abundances of inflammation-related *Solobacterium* and *Oribacterium*, the relative abundance of Bifidobacterium increased markedly, changes of BRANS total score, including 5 domains such as immediate memory, visual–spatial, language, attention, and delayed memory	older adults
Boehme et al. (72)	*Bifidobacterium longum* NCC3001	Stress relief	Improved sleep quality, reduction in anxiety, depression, and cortisol awakening response	adults
Morales-Torres et al. (73)	*Lactobacillus helveticus* R0052 and *Bifidobacterium longum* R0175	No significant effect	No significant effect	adults
Lee et al. (76)	*Lactobacillus reuteri* NK33 *and Bifidobacterium adolescentis* NK98	Mental health and sleep	Reduced depressive symptoms, anxiety, and improved sleep quality; increased *Bifidobacteriaceae* and *Lactobacillacea*	adults
Huang et al. (77)	*Lactobacillus plantarum* TWK10	Exercise performance	Decreased body fat significantly and increased muscle mass significantly	adults
Aida et al. (82)	*Heyndrickxia coagulans strain* SANK70258	Upper respiratory tract infection	Induced anti-inflammatory effects (decreased IL-6 and TNFα level) via increased intestinal butyrate levels	adults
Altadill et al. (83)	*Lactoplantibacillus plantarum* DR7	Upper respiratory tract infection	Reduced the proportion of patient days of URTI and of fever	adults
Bertuccioli et al. (84)	*Streptococcus salivarius* K12	No effect for upper respiratory tract infection	Increased sIgA levels	adults
Garvey et al. (87)	*Bacillus subtilis* BS50	Gastrointestinal symptoms	Improvement of burping and bloating	adults

Since there is increasing evidence that exercise can modify gut microbiome profile, the following chapters focus on the other perspective, an overview of current knowledge from the past 5 years regarding exercise performance changes related to probiotics and different types of exercise in humans.

## 4 Endurance-based sports

The study investigated road cyclists who were supplemented with probiotics, and it demonstrated that there are no effects on the body composition of the athletes, except for an elevated muscle mass after a 4-month supplementation period. Furthermore, the long-term probiotics supplementation increased levels of the athlete’s aerobic capacity and positively affected the oxidative stress markers such as total oxidative status (TOS), TNF-*α* (tumor necrosis factor-alpha), and IL-6 (interleukin 6) related to the exercise capacity of competitive road cyclists (88). The elite road cyclist who received a multi-strain probiotic supplementation for 90 days reported a significantly lower incidence of GI symptoms compared to the placebo group, mean rate of perceived exertion values during the TTF (time-to-fatigue) was lower in the supplemented group, however, the authors did not find differences in VO_2max_ and TTF values (89).

Eight weeks of *Bifidobacterium lactis* BL-99 administration showed an improvement in lipid metabolism markers such as DHA (docosahexaenoic acid), adrenic acid, linoleic acid, and acetic acid, and decreases in glycocholic acid and glycodeoxycholic acid; furthermore, the probiotics supplementation improved the VO_2max_ and the knee-joint extensor strength in cross-country skiers (90).

URTIs in runners can present symptoms like a sore throat, coughing, congestion, a runny nose, mild fever, and fatigue. These infections, often caused by viruses like the common cold, can impair breathing, reduce oxygen uptake, and increase perceived exertion, making it harder for athletes to train or compete effectively. For athletes, especially endurance runners, URTIs are significant because even mild symptoms can lead to a decline in performance. A 6-week intervention with *Lactobacillus helveticus* Lafti L10 did not change time to exhaustion and the GI and cold/flu-like symptoms in exercise endurance among non-elite athletes (91). In contrast, the URTI symptoms were lower before the marathon, and the multi-strain probiotic administration affects cytokine production by monocytes after the competition in runners (92). In a longer supplementation study, 3 months of multi-strain probiotic administration increased serum HDL cholesterol and decreased LDL cholesterol and triglyceride levels, and the runner participants reported an improvement in general health (93). In endurance runners, a probiotic cocktail containing *P. acidilactici* and *L. plantarum* proved to be safe and did not affect gut or immune-associated parameters or intestinal symptoms after 4 weeks of supplementation (94). In long-distance male runners, an increase in lean body and skeletal muscle mass was demonstrated, while in the group of women taking the multi-strain probiotic for 1 month, a decrease in the content of total body fat and visceral fat was observed and the VO_2max_ increased in both the women and men (95). The other study, which investigated long-distance runners, demonstrated that the *Bifidobacterium longum subsp. longum* Olympic No. 1 supplementation for 5 weeks significantly changed the 12-min Cooper’s test running distance and the abundance of gut microbiota (96). In marathon runners, pro-inflammatory cytokine production by stimulated lymphocytes decreased after 30 days of probiotic supplementation (97). We propose an overview of the probiotics, dosage, duration of supplementation, and their effects on performance in Table 3.

**Table 3:** Summary of probiotic strains, dosing strategies, supplementation duration, and their reported effects on performance in endurance-based sports.

Authors	Sport	Probiotic supplementation	CFU	Duration	Effects for performance
Batatinha et al. (97)	Running	*Bifidobacterium-animalis-subsp.-Lactis, Lactobacillus-Acidophilus*	10 × 10^9^	30 days	↓ pro-inflammatory cytokine production, maintained CD8 T cell and effector memory cell population
Lennon et al. (94)	Running	*Pediococcus acidilactici, Lactobacillus plantarum*	3 × 10^9^	4 weeks	No effects for GI symptoms
Lin et al. (96)	Running	*Bifidobacterium longum subsp. longum* OLP-01	1.5 × 10^10^	5 weeks	↑ running distance ↑ gut microbiota abundance
Mazur-Kurach et al. (88)	Road cycle	*Lactobacillus plantarum, Lactobacillus casei, Lactobacillus rhamnosus, Bifidobacterium breve, Lactobacillus acidophilus, Bifidobacterium longum, Bifidobacterium bifidum, Bifidobacterium infantis, Lactobacillus helveticus, Lactobacillus fermentum, Lactobacillus bulgaricus, Lactococcus lactis, Streptococcus thermophilus*	1 × 10^11^	4, 12 and 16 weeks	↑ level of aerobic capacity ↑ magnitude of maximal oxygen uptake ↑ duration of exercise to failure ↓ heart rates
McDermott et al. (91)	Running	*Lactobacillus helveticus Lafti* L10	5 × 10^9^	6 weeks	↓ time-to-exhaustion
Schreiber et al. (89)	Cycle	*Lactobacillus helveticus Lafti* L10, *Bifidobacterium animalis ssp. lactis Lafti* B94, *Enterococcus faecium* R0026, *Bifidobacterium longum* R0175, *Bacillus subtilis* R0179	4.3 × 10^9^ ≥4.3 × 10^9^ ≥3.9 × 10^9^ ≥2.1 × 10^9^ ≥0.4 × 10^9^	90 days	↓ GI symptoms No significant effect in VO_2max_ and time to fatigue
Smarkusz-Zarzecka et al. (95)	Running	*Bifidobacterium lactis* W52, *Lactobacillus brevis* W63, *Lactobacillus casei* W56, *Lactococcus lactis* W19, *Lactococcus lactis* W58, *Lactobacillus acidophilus* W37, *Bifidobacterium bifidum* W23 and *Lactobacillus salivarius* W24	2.5 × 10^9^	12 weeks	↑ VO2_max_ ↑ minute ventilation ↑ functional capacity ↑ breathing reserve ↑ exercise capacity
Smarkusz-Zarzecka et al. (93)	Running	*Bifidobacterium lactis* W52, Levilactobacillus *brevis* W63, *Lactobacillus casei* W56, *Lactococcus lactis* W19, *Lactobacillus lactis* W58, *Lactobacillus acidophilus* W37, *Bifidobacterium bifidum* W23, *Ligilactobacillus salivarius* W24	2.5 × 10^9^	12 weeks	↓ GI symptoms
Li et al. (90)	Skiing	*Bifidobacterium lactis* BL-99	1 × 10^9^	8 weeks	↑ SCFAs level ↑ PUFAs level ↑ bile acids ↑ 180°/s knee joint extensor strength ↑ 60°/s knee joint extensor strength ↑ VO_2max_
Tavares-Silva et al. (92)	Running	*Bifidobacterium-animalis-subsp.-Lactis, Lactobacillus-Acidophilus*	10 × 10^9^	30 days	↓ pro-inflammatory cytokine production ↑ numbers of naïve CD8 + T cells

## 5 Intermittent exercise-associated sports

Salleh et al. (98) presented that 6 weeks of *Lactobacillus casei* supplementation improved aerobic capacity and reduced anxiety and stress in badminton players. Multi-strain probiotics in combination with dietary fiber for 23 days showed a reduction in inflammatory process activity and peripheral blood lymphocyte apoptosis in basketball athletes (99).

In professional soccer players, 1 month of synbiotic administration improved physical activity, sleep quality, and perceived general health, stress, and anxiety levels. Furthermore, the synbiotics induced an immunophysiological bioregulatory effect in the athletes (100). The *Lactobacillus casei* Shirota strain showed positive effects on anxiety-induced physiological parameters in football players (101). 6-week synbiotics supplementation, which is a combination of probiotics, significantly reduced the URTI symptoms, the incidence, and the duration of the symptoms in football players. In addition, the HR_max_ (maximal heart rate) and ER (lactic acid elimination rate) were markedly increased compared to the basal level during the recovery period after exercise in the symbiotic-supplemented group (102). In contrast, there were no significant changes in pain and fatigue in dancers after 12 weeks of *Lactobacillus helveticus* Rosell-52 and *Bifidobacterium longum* Rosell-17 supplementation (103). Furthermore, the probiotic mix capsule (containing eight different strains) did not change the TTE (time-to-exhaustion), RCP (respiratory compensation point), time-HHV (hypocapnic hyperventilation area time), and VO_2max_ levels after 4 weeks in soccer players (104). We propose an overview of the probiotics, dosage, duration of supplementation, and their effects on performance in Table 4.

**Table 4:** Summary of probiotic strains, dosing strategies, supplementation duration, and their reported effects on performance in intermittent exercise-associated sports.

Authors	Sport	Probiotic supplementation	CFU	Duration	Effects for performance
Adikari et al. (101)	Football	*Lactobacillus Casei* Shirota strain	3 × 10^10^	8 weeks	↑ cognitive test reaction time (digit vigilance test)
Imanian et al. (104)	Soccer	*Lactiplantibacillus plantarum* BP06, *Lacticaseibacillus casei* BP07, *Lactobacillus acidophilus* BA05, *Lactobacillus delbrueckii* BD08 *bulgaricus*, *Bifidobacterium infantis* BI04, *Bifidobacterium longum* BL03, *Bifidobacterium breve* BB02 and *Streptococcus salivarius thermophilus* BT01	4.5 × 10^11^	4 weeks	No significant effect in TTE (time-to-exhaustion), RCP (respiratory compensation point), time-HHV (hypocapnic hyperventilation area time), and VO_2max_ levels
Zhang et al. (102)	Football	*Lactobacillus casei Zhang*, *Bifidobacterium lactis* V9, *Lactobacillus plantarum* P-8	≥8 × 10^9^ ≥8 × 10^9^ ≥6 × 109	6 weeks	↓ URTI symptoms ↑ SIgA level ↓ inflammatory factors ↓ HR_max_ ↓ ER (Lactic Acid Elimination Rate)
Quero et al. (100)	Soccer	*Bifidobacterium lactis* CBP-001010, *Lactobacillus rhamnosus* CNCM I-4036, *Bifidobacterium longum* ES1	≥1 × 109	1 month	↓ stress level ↓ anxiety level ↓ depression level ↑ post-exercise dopamine concentration
Salleh et al. (98)	Badminton	*Lactobacillus casei* Shirota	3 × 10^10^	6 weeks	↓ stress levels and anxiety ↑ aerobic capacity
Trushina et al. (99)	Basketball	10 of probiotic strains of *Bifidobacteria and Lactobacilli*	≥1.25 × 1,010	23 days	↓ inflammatory process activity ↓ peripheral blood lymphocyte apoptosis
Wiącek et al. (103)	Dance	*Lactobacillus helveticus* Rosell-52, *Bifidobacterium longum* Rosell-17	3 × 10^9^	12 weeks	No significant changes in pain and fatigue

## 6 Resistance training-associated sports

Research on strength and power-based sports within this field is currently limited, resulting in a scarcity of published articles addressing this type of sport. In resistance-trained males, 30 and 60 days of *Bacillus coagulans* Unique IS-2 supplementation significantly increased the BCAA absorption and improved leg press and vertical jump power (105). In addition, *Bacillus subtilis* supplementation (5 billion CFU/day) improved the body composition of Division I female athletes in the 10-week resistance training program, where the athletes completed 3–4 workouts per week of upper- and lower-body exercises and sport-specific training (106). This study observed significant effects for improved squat, deadlift, and bench press 1 repetition maximum. The body composition analysis showed changes in body fat, muscle thickness, and rectus femoris after 10 weeks of supplementation. In rugby players, a shorter, 17-week daily probiotics (*Lactobacillus acidophilus*, *Bifidobacterium bifidum*, *B. animalis ssp. Lactis*) administration improved sleep quality and decreased muscle soreness and leg heaviness scores (107).

## 7 Wheelchair athletes

Gut disorders are a major contributor to morbidity among wheelchair athletes. The impact of probiotics on these athletes is not well-published. Nevertheless, there is only evidence of whether probiotics can improve the health and quality of life of wheelchair athletes. After a 12-week of probiotic administration, it has been confirmed that probiotics decreased inflammatory markers and improved the diversity of the gut microbiome (108). The probiotic contained *Bifidobacterium lactis* W51, *Bifidobacterium lactis* W52, *Enterococcus faecium* W54, *Lactobacillus acidophilus* W22, *Lactobacillus paracasei* W20, *Lactobacillus plantarum* W21, *Lactobacillus salivarius* W24, *Lactococcus lactis* W19. On the other hand, a total of 8 weeks of freeze-dried multispecies probiotic *Bactosan pro FOS* supplementation did not significantly reduce the GI symptoms (109).

## 8 Discussion

As we know, many factors affect the physiological performance of athletes, namely infections (i.e., URTIs), circadian rhythm, cognitive functions, stress, mental health, inflammatory responses, changes in skeletal muscle or body lean mass, gastrointestinal symptoms, etc. Technological advances have expanded knowledge of the gut microbiome from earlier beliefs. The topics covered in this review are new perspectives of what is currently understood regarding how the human gut microbiome changes after exercise and probiotics, and how probiotic administration impacts sports performance-related factors. The role of the endocrine and nervous routes in improving sleep efficiency was explained by previous studies that have reported the influence of probiotics on psycho-neuro-endocrine-immune activity via signals from the GI tract (110). Certain strains of bacteria produce substrates such as lactate and acetate for butyrate production, and it was associated with better cognitive function via modulation of the gut-brain axis (111, 112). Ameliorated lipid metabolism through SCFAs and PUFAs might have resulted from activated AMPK signaling pathway, which modulates fat synthesis. Another potential perspective is that a combination of exercise and probiotics has a positive effect on intestinal barrier function and systemic inflammatory response. These signaling pathways can amplify the gut-liver axis, which regulates metabolites in the intestinal area (113). Both lipid metabolism and the gut–liver axis are linked to athletic performance. Previous studies demonstrated that the gut–liver communication can significantly influence energy availability, inflammation, and recovery processes in human participants (114, 115). In addition, the probiotic supplementation might improve glucose metabolism, anti-inflammatory signaling activities through immunomodulation, and the balance of microbiome in the gut, thereby promoting an increase in VO_2max_ (116). Optimizing probiotic intake, strains, timing (i.e., duration), and amount (colony-forming units, CFU) can help athletes improve their physiological performance. The impact of probiotics on athletes is a relatively new research field, with only a small number of studies conducted so far. While these studies have shown significant potential, there is still a limited understanding of the benefits of probiotics for highly active individuals and whether they gain from them.

## 9 Conclusion

Importantly, the effectiveness of probiotic supplementation on performance-related outcomes such as sleep quality and quantity, well-being, concentration, power, quickness, GI symptoms, heart rate, etc. is determined by multiple factors, including the specific probiotic strains and species, the colony-forming units (CFU), and the dosage protocol and strategy—encompassing both the duration and frequency of intake. These variables may influence the physiological impact of the supplementation either independently of, or in interaction with, exercise protocols, and might result in different effects. Furthermore, the positive effects depend on the type of sports. Probiotics show promise for enhancing both endurance and intermittent exercise performance, primarily through mechanisms such as the reduction of gastrointestinal symptoms and systemic inflammation. These effects may contribute to enhancing immune function, nutrient absorption, and overall performance and training capacity. However, current evidence supporting their efficacy in resistance-based sports remains limited and inconclusive. To establish more definitive conclusions, future research could focus on standardizing methodologies and assessing the physiological impacts of probiotics on elite athletes, including wheelchair athletes, focusing on consistent protocols and strain-specific interventions. Learning more about how probiotics and the microbiome change in exercise performance may provide new insights for new training techniques and strategies for professional athletes.
